# The Automatic Methods Group *Newsletter*

**DOI:** 10.1155/S1463924695000241

**Published:** 1995


Journal of Automatic Chemistry, Vol. 17, No. 4 (July–August 1995), pp. 163–168

AUTOMATIC METHODS GROUP

Analytical Division
ROYAL SOCIETY OF CHEMISTRY

### The Automatic Methods Group *Newsletter*

The AMG Newsletter is published as part of and in collaboration with the *Journal of Automatic Chemistry*. Further information on the *Journal of Automatic Chemistry* can be obtained from Taylor & Francis Ltd, Rankine Road, Basingstoke, Hampshire RG24 8PR or e-mail: info@tandf.co.uk; worldwide web home page: http: ||tandf.co.uk.

### An Introduction to the Automatic Methods Group

*'I wish to suggest that Council (of the Society of Analytical Chemistry) should consider the possibility of funding a Group or Panel on automatic analysis'.*

This suggestion, in a letter dated 8 November 1963, from Dr Jim Page to the President of the SAC, Dr D. C. Garrett, led, in 1965, to the creation of the Automatic Methods Group. From the first, the Group's remit was to provide a forum for contact for those members of the Analytical Division who, either through interest, or perforce through the demands of ever-increasing workload, were acquiring, leasing, or in some cases building, automatic analysers.

For 30 years the Automatic Methods Group has sought to inform and educate members of the Royal Society of Chemistry on aspects of automation. The AD's council has seen fit to extend its original remit, first to include process control, in recognition of the close ties between analyses and their applications, and later to LIMS as it became clear that analysis alone was only half the story—to be effective, chemical measurements had to be communicated back to the requester in ever shorter timescales.

Drawing much of its membership from industry, the Automatic Methods Group also has a role to play in influencing the next generation of instruments, as its deliberations are fed back to analyser manufacturers, and international standardization bodies.

Yet, despite the seminars and meetings, one question remains—Jim Page's letter again from 1963—'a symposium on automatic methods . . . was exceptionally well attended (*but not by S.A.C. Members*)'. Around half the delegates at our meetings are neither RSC nor AMG members. If you are a member, but we are not holding your kind of meeting, tell us so! And if we are holding your kind of meeting, and you are not a member, wouldn't you like to join us?

Dr. A. S. McLelland

### Meeting Reports

### The air we breathe—The use of automation in indoor, ambient and workplace monitoring

*The Automatic Methods Group of the Royal Society of Chemistry held a joint meeting with the Health and Safety Executive on 14 December 1994 at The Scientific Societies Lecture Theatre, London W1. Sponsored by M-Scan; PS Analytical; Perkin Elmer; and Reading Scientific Services, the meeting was chaired by Dr Kevin Saunders of Keris Ltd and Dr Alan Braithwaite of Nottingham Trent University. Presentations included: 'The new European legislation and the impact of standardization, by Dr Richard Brown, Health and Safety Executive, Sheffield; 'AQUACHECK, accreditation and proficiency scheme, by Dr Ian Taylor, Aquacheck, WRc Medmenham; 'Monitoring emissions from building materials' by Dr Derek Crump, Building Research Establishment, Garston; 'Automated techniques for air monitoring' by E. J. Hoekstra, R. J. B. Peters, Dr Ed DeLeer, TNO Environmental and Research Institute, The Netherlands; 'Current problems in workplace monitoring' by Dr Steve Lewis, BP International, Sunbury on Thames; 'Interface of ambient, indoor and workplace particulate monitoring' by Dr David Mark, AEA Technology, Harwell; 'The characterization and measurement of malodours' by Dr Robert Large, M-Scan, Ascot; 'Automated methods for aerosol monitoring' by Dr Lee Kenney, Health and*

0142-0453/95 $10.00 © 1995 Taylor & Francis Ltd.

*Safety Executive, Sheffield; and 'Measurement of mercury in ambient air' by Dr Peter Rabl, Bayerisches Landesamt für Umweltschutz (Bavarian State Office for Environmental Protection), Munich, Germany. Abstracts of some of the papers are included in this newsletter for readers who did not attend.*

### Abstracts of papers presented

### The new European Legislation and the impact of standardization

**Richard H. Brown**
*Environmental Measurement Group, Health and Safety Laboratory, Health and Safety Executive, Broad Lane, Sheffield, UK*

Two important Directives are being developed in Europe: a Chemical Agents Directive and an Ambient Air Directive. The first concerns the workplace and the second outdoor air, but there are also legislative requirements or guidance values on indoor air quality and on particular emissions, such as from stacks or automobile exhausts. The new Chemical Agents Directive is a sort of 'EuroCOSHH' and, if implemented, will give general provisions for protecting workers from the effects of chemical agents in the workplace. The new Ambient Air Quality Directive, if implemented, will establish objectives for ambient air quality throughout Europe. Both Directives have a secondary objective to improve air quality measurement, so that provisions are included to link reliable analytical methodology to the air quality objectives. In particular, the Chemical Agents Directive refers back to Directive 88/642/EEC, where an annex specifies that the 'reference' methods to be used in its implementation should conform to guidelines promulgated by CEN or ISO. It also contains provisions for a review within five years to ensure that the methods comply with the 'general requirements for the performance of measuring procedures and devices for workplace measurements'. This is almost exactly the title of a standard (EN482) developed by CEN/TC137, which was set up in 1988 in order to provide the technical input implied by the workplace Directives. Similar technical input has been provided for the ambient air Directives through CEN/TC264, although here the activity has been mainly in developing specific methodology. These developments have clear implications for measurement strategies both locally and across Europe. The existence of European Directives encourages the European Commission to promote research in related measurement areas, particularly through the DGXII Measurement and Testing (M&T) Programme. Thus, currently supported projects include the investigation of sampling errors (the so-called 'Mol' intercomparisons), a study on sorbing agents for VOCs and a pilot study on the testing of workplace aerosol sampling instruments to CEN particulate fraction definitions. More directly, EN482 leads to a requirement for testing the available measurement methods, and, if they do not comply with the CEN performance criteria, develop new ones. An initiative is under way, therefore, to propose a project under the 4th M&T Programme which will undertake this research. Closer to home, individuals or companies wishing to use measurement methods in support of Directives or local legislation will also need to take the provisions of the Directives and the CEN norms into account, and ensure that the methods that they use are valid. Look to your SOPs!

### AQUACHECK, accreditation and proficiency scheme

**Ian Taylor**
*Aquacheck, WRc-Medmenham Medmenham, Marlow, Buckinghamshire SL7 2LD, UK*

The implementation of European Community Directives with the associated Environmental Quality Standards has placed greater emphasis on the maintenance of water and waste quality to meet specific numerical standards. In order to ensure that the results they obtain are adequately accurate, laboratories now require highly developed analytical quality control (AQC) systems, external accreditation and external monitoring of analytical capability. In 1985 the WRc initiated the AQUACHECK proficiency testing scheme to meet the demand for external monitoring of analytical performance.

The AQUACHECK scheme aims:

(1) To provide a continuing check on the accuracy and comparability of analytical data.
(2) To identify the determinands for which improved accuracy is required.

The scheme is split into 25 determinand packages, including metals, nutrients, organics and non-specific determinands. Seven matrices are covered, including freshwaters, wastewaters, saline waters, soils and sewage sludges. A laboratory may enter the scheme for some or all of these groups.

AQUACHECK currently covers the following sample matrices and determinand groups:

(1) Drinking water (major constituents, nutrients, non-specific determinands, trace metals and toxic trace metals).
(2) Wastewaters (nutrients, non-specific determinands and metals).
(3) Industrial wastewaters (settled COD, pH, suspended solids, total phenol, cyanide, sulphate, ammonia, SRP and metals).
(4) Organics in cleanwater and wastewater (haloforms, phenols, organochlorine pesticides, chlorinated solvents, PAHs, PCBs organophosphorus pesticides and herbicides).
(5) Saline waters (nutrients and metals).
(6) Sewage sludge (metals, N, P, K, F).
(7) Soil (metals, N, P, K, F).

AQUACHECK covers a broad participant base—clients include the major water utilities, NRA, DoE, River Purification Boards, Scottish Regional Councils and a variety of commercial laboratories. AQUACHECK's geographic spread gives a significant international dimension to its comparison of laboratory quality (see table 1).

*Table 1. Number of Participants (by location) in AQUACHECK.*

| | | | |
|---|---|---|---|
| United Kingdom | 167 | Belgium | 46 |
| Czech Republic | 14 | Ireland | 11 |
| Spain | 5 | Germany | 3 |
| Indonesia | 1 | Italy | 1 |
| Denmark | 1 | Sultanate of Oman | 1 |
| Hong Kong | 1 | New Zealand | 1 |
| Total (September 1993) | 252 | | |

### Monitoring emissions from building materials

**D. R. Crump**
*Building Research Establishment, Watford, Herfordshire, UK*

Studies of indoor air quality in all types of buildings throughout the developed world have shown the presence of hundreds of organic compounds at concentrations exceeding those in outdoor air. There is concern that these compounds can cause discomfort to occupants through sensory effects of the type associated with 'Sick Building Syndrome' and that they are a significant route of exposure to a range of genotoxic and carcinogenic chemicals.

The sources of organic compounds within a building are numerous and can be categorized as (1) outdoor air; (2) materials for construction and furnishing; (3) combustion of fuels; (4) use of consumer products by occupants, including environmental tobacco smoke (ETS). The relative importance of these sources varies according to individual compounds, building characteristics and location, and occupant behaviour. For example, concentrations of formaldehyde in homes in England are typically 12 times higher than those found outdoors because of the many sources of formaldehyde in the indoor environment, such as particleboard. This contrasts with benzene where the indoor concentration is typically 1·3 times the outdoors, and the main source is emissions from motor vehicles.

Occupant dissatisfaction with air quality in homes and offices has led to considerable pressures to minimize the emission of organic compounds from materials used in buildings. Control of these emissions requires a means of characterizing the release, and environmental test chambers in a variety of forms have been developed for this purpose. The principle of these tests is to enclose a sample of the material within a stainless-steel enclosure supplied with clean and conditioned air. The material loading (surface area to volume of chamber) is selected as being typical of a room situation. The organic compounds in the air leaving the chamber are determined and used to calculate the emission rate for each compound of interest.

The European Standards Organization (CEN) is currently drawing up standard procedures for these tests and these are expected to become a requirement for demonstrating compliance with the Construction Products Directive (CPD). This, together with the existing voluntary schemes and regulations being applied in other countries, is responsible for a fast-growing interest in the monitoring of emissions from building materials.

### The contribution of natural emissions to air quality: automated and automatic sampling

**E. J. Hoekstra, R. J. B. Peters and Ed. W. B. de Leer**
*TNO Institute of Environmental Sciences, Delft, The Netherlands*

Naturally produced compounds, such as terpenes and some organohalogen compounds, contribute significantly to the total emission of hydrocarbons and organohalogen compounds to the atmosphere. These naturally produced compounds thereby contribute to atmospheric quality problems, such as summer smog formation in the troposphere and the degradation of the stratospheric ozone layer.

The determination of natural emissions requires a special analytical approach. The emission of terpenes from a forest can be calculated from the terpene concentration gradient above the forest canopy. This requires simultaneous sampling of terpenes at different heights up to about 30 m above the canopy. Automated sampling on Tenax TA tubes, followed by analysis with thermodesorption GC-ITD, was used to solve these problems. The analytical procedure was described, together with the validation procedure. Results from a sampling campaign in The Netherlands were presented.

The natural production of organochlorine compounds in soil top layers has received considerable attention in the past five or six years. Chloroform is one of these naturally produced compounds, which diffuses from soil and contributes to the background chloroform concentration in the atmosphere.

The total emission rate from soil was determined by covering the soil with a closed container. The chloroform was collected in the container on a diffusive sampler, which acted as an automatic sampling device. The sampler uptake was described with a mathematical model which indicates that a 14-day sampling period collects about 90% of the total emission. The chloroform was analysed by thermodesorption GC-MS.

### Current problems with workplace monitoring

**Steve J. Lewis**
*BP International, Sunbury on Thames, UK*

There are several facets of a workplace monitoring exercise including: review of general requirements; selection of methods; selection of strategy; preparations for monitoring; transport to and from sampling site; and on-site sampling.

Prior to any survey work, it is necessary to set down the objectives of the monitoring exercise as this will influence the approach that is adopted. It is necessary to review the site activities and materials to ensure that the correct substance is targeted. On occasions, requests for sampling do not focus on the substance that is likely to present the greatest health risk.

### *Methods*

Chemical substances can be present in the atmosphere in a gaseous, liquid or solid state and as a consequence different techniques have to be adopted for sampling. There are several hundred sampling and analytical methods published by different organizations and these include the Methods for the Determination of Hazardous Substances (MDHSs) published by the Health and Safety Executive.

*Strategy*

Several strategies may be adopted and these can include sampling for different periods, for example peak, short term, partial shift and full shift. In addition, measurements can be relatively crude or extremely sophisticated. The strategy chosen must be appropriate to the objectives of the monitoring.

*Preparation*

All equipment must be properly maintained and calibrated and requires careful preparation to avoid errors. Additional equipment may be required in case of breakdown and suitable ancillary equipment including tools, sampling sheets and equipment manuals should be taken to the sampling locations.

*Transport*

Suitable transport procedures must be adopted to avoid damage to equipment and contamination of collection devices.

*On-site sampling*

There are many problems that can arise during the sampling exercise, including sampling equipment set up, deployment and failure, process and personnel difficulties. In order to minimize problems, careful planning, preparation and execution of sampling is necessary.

### The interface of ambient, indoor and workplace particle monitoring

**David Mark**
*Aerosol Science Centre, B401.8, AEA Technology, Harwell, Didcot, Oxfordshire OX11 0RA, UK*

In a limited survey of my friends, acquaintances and colleagues, it was found that on average, working people spend 25% of their time at work, 6% travelling, 5% on outside activities, 30% asleep and the remaining 34% at home eating and relaxing etc. For housewives/house husbands the proportions were 10% outside the home, 30% sleeping and 60% at home doing the housework, cooking etc. During all this time there is the potential for exposure to airborne particles.

However, in the UK, exposure measurements have been concentrated mainly in the workplace, to minimize deaths and ill health from deep lung diseases such as silicosis, pneumoconiosis, byssinosis, etc. As Health and Safety at Work legislation has been increasingly applied, the airborne dust levels in workplaces has gradually reduced to the extent where the workplace may not be the major source of particulate dose. In the ambient atmosphere, despite a large number of deaths associated with the inhalation of smoke and $SO_2$ in the London smogs of the early 1950s, very little relevant particle sampling has been carried out. Even less particle sampling has been carried out indoors in homes where people spend most of their time.

This paper reviewed the strategies for particle sampling in all environments where people spend their time and presented some ideas for an integrated measurement programme. This is particularly timely in the light of the current concern over the increase in the incidence of asthma in the general public. Both outdoor (increased levels of traffic fumes from rapidly increasing numbers of diesel and petrol-driven vehicles) and indoor (for example particles from pets and mould spores) have been implicated in the aetiology of the disease.

### The characterization and measurement of malodours

**Robert Large, Christopher A. J. Harbach and Peter J. C. Tibbetts**
*M-Scan Ltd, Silwood Park, Sunninghill, Ascot, Berkshire SL5 7PZ, UK*

Society increasingly expects a clean environment, particularly the air we all breathe. In fact, the first indication of a potential environmental problem is often the detection of a malodour associated with a particular plant, activity or process. The malodour may not in itself present a direct risk to human health, but, nevertheless, may constitute an unacceptable nuisance to people living in the vicinity of the activity in question.

The individual chemical compounds responsible for such malodours are often present in the atmosphere at low ppb concentrations in the presence of much higher concentrations of irrelevant, non-malodorous compounds. This places stringent demands on the chemical analyst who is expected to detect proverbial needles in haystacks. Methods developed for use in the safety and occupational hygiene fields normally require percentage level and ppm level sensitivity, respectively; such methods are inappropriate for the specific analysis of malodours, particularly at very low chemical signal-to-noise ratios.

The limited range of methods available for the characterization and measurement of malodours were reviewed and illustrated by reference to particular case studies.

The particular procedure optimized by M-Scan involves collection of organic vapours, including those responsible for the malodour, onto a suitable adsorbent tube and individual analysis by thermal desorption/cryogenic trapping/flash vaporization/capillary GC/MS using thick film GC columns to maximize resolution. A prior knowledge of the type of smell involved, for example, fishy, sulphurous, sweaty socks, allows the huge array of recorded GC/MS data to be searched mass chromatographically for candidate compounds, for example amines, mercaptans/thioethers, acids/aldehydes. The detection of individual compounds is confirmed by library matching and inspection of the individual mass spectrum and GC retention time. Malodorous compounds are normally volatile and chemically simple with few isomers. The prospects of positive analysis of malodours are therefore normally good.

### Automated methods for aerosol monitoring

**Lee C. Kenny**
*Health and Safety Laboratory, Broad Lane, Sheffield S3 7HQ, UK*

There is a very large number of instruments available commercially for automated monitoring of aerosol properties, covering a wide range of applications. The output of these instruments is generally expressed as aerosol number concentration, mass concentration or particle size distribution; these quantities are obtained via calibration from measurements of the gravimetric, optical, aerodynamic, mechanical, or electrical properties of the aerosol particles. Depending on the application, the instrument's operating range in terms of particle size and concentration may be optimized within limits imposed by the technology. The accuracy, precision, linearity and stability of the calibration, under the conditions of use of the instrument, determines whether the instrument's readings are quantitative, semi-quantitative or merely indicative.

The most widely used automated aerosol monitors can be divided into two classes, those using dynamic mass measurement, and those using optical light scattering or extinction. Dynamic mass instruments use extractive sampling, and quantify mass using either beta-attenuation, piezoelectric or tapered-element oscillations. Optical instruments may use either extractive or in-situ measurement, and may measure either single particles or assemblies of particles. The key components of all these instruments are an aerosol sampling system (for extractive instruments), a sensing zone and/or collection substrate, and a data processing module. Some of the characteristics and limitations of typical instruments will be described.

Instruments intended for use in clean-room monitoring, ambient or indoor air quality monitoring or occupational hygiene are required to meet the different standards that apply in those fields. The standards and performance requirements for instruments used for health-related monitoring of aerosols in workplaces are difficult to apply to the automated aerosol monitors that are currently available. This is principally because the limited nature of laboratory calibration means that measurements in workplaces are usually only semi-quantitative. However, semi-quantitative automated instruments have many useful applications in occupational hygiene, some examples of which will be described. Techniques for field calibration of instruments have also been developed, and these can potentially improve the quality of measurements made with automated aerosol monitors.

### Measurements of mercury in ambient air

**Peter Rabl and Martin Kretschmann**
*Bayerisches Landesamt für Umweltschutz (Bavarian State Office for Environmental Protection), Munich, Germany*

In a closed chemical factory in northern Bavaria mercury (Hg) polluted soil (mercury content up to one percent) was dredged and cleaned under commission of the Bavarian State Ministry of State Development and Environmental Affairs.

In order to control the ambient air pollution during this work, monthly measurements of Hg deposition in and around the polluted area were performed. The concentration of gaseous Hg matter in the air was monitored continuously with an automatic device in the immediate area, and downwind at irregular intervals. To determine the Hg deposition, 1 litre PE breakers, supplied with 100 ml of a 4% $K_2Cr_2O_7$ solution, were exposed for one month to the atmosphere and were then analysed by AAS or UV fluorescence. For automatic irregular and continuous Hg measurements, air samples were sucked through small quartz tubes containing gold-covered quartz powder or platinum nets. Mercury is absorbed as amalgam on the probes. After thermal decomposition of the amalgam ($T > 700°C$), the desorbed elementary Hg was quantified by a sensitive UV fluorescence detector. It is possible to distinguish between elementary and organic Hg matter by means of a special supplementary charcoal filter.

Close to the polluted area, the ambient air concentration of Hg depended to a great extent on temperature—the values were highest in summer. There was hardly any correlation between Hg deposition and the seasons or the deposition of particulate matter. Hg concentration in air and Hg deposition decreased rapidly with increasing distance from the source. Basic ambient levels were observed downwind up to 0.5 to 1 km away from the area.

There were Hg concentrations up to some 10 $\mu g/m^3$ in the polluted area, but the WHO guideline value (annual average) of 1 $\mu g/m^3$ was not exceeded outside the area. The annual mean values of Hg deposition were usually less than 1 $\mu g/m^2d$ in the surrounding area, whereas single monthly values of up to 30 $\mu g/m^2d$ were observed. There was clearly a relationship between Hg deposition and Hg content of food plants, growing in and around the polluted area.

---

### Forthcoming AMG Programme

| Date | Title | Venue |
|---|---|---|
| 7 Dec 1995 | Robotics and Automation in the Pharmaceutical Laboratory (Held in conjunction with the Joint Pharmaceutical Analysis Group) | London |
| 12–13 Dec 1995 | Environmental Monitoring III: Monitoring for the Needs of Society: New Horizons (Held in conjunction with the South East Region of the AD) | London |

Further information and other meetings from the Secretary, D. G. Porter, Willowfold, Fir Tree Lane, West Chiltington, West Sussex RH20 2RA (tel: 01798 812383).

### Forthcoming Meeting

### The industrial/academic interface

**A joint meeting between the Western Region and the Automatic Methods Group, Robbins Conference Centre, University of Plymouth**
**Thursday 7 September 1995**

In an increasingly value-conscious culture analytical scientists are being driven to find novel means of developing cost effective measurement solutions, particularly in the realms of automated analyses.

Often the inability to effectively address these issues in-house stems from lack of resource or skill base. These issues are now providing the stimulus for many organizations to seek outside input at levels which would not have been considered a decade ago. As a result an increasing number of organizations have been actively extending team working externally to include academic input.

From these joint activities, it is becoming increasingly apparent that successfully planned and managed projects are providing the means for developing novel, cost effective, measurement solutions.

Interestingly, another significant benefit of these endeavours lies in the increased understanding of mutual needs. This is creating an awareness that the lasting benefit of such joint ventures lies in the opportunity such projects provide to develop and transfer *skills* rather than *technology*.

This meeting will cover all aspects of project management in such a joint venture and will be of interest to laboratory managers, scientists and their customer base. It will discuss a range of programmes involving automated instruments. The availability of government sponsorship for such projects will also be reviewed.

### Programme

| | |
|---|---|
| 11.30 | Registration |
| 12.15 | Lunch (provided) |
| 13.30 | Chairman's Introduction |
| 13.40 | The Industrialist's View<br>Don White, BP Research, Sunbury |
| 14.05 | Overview of Teaching Company Scheme<br>Roland Burns, Plymouth Teaching Company Centre |
| 14.30 | The Academic's View<br>Professor Les Ebdon, University of Plymouth |
| 14.55 | The Student's View<br>Noel Brahma, Teaching Company Associate at P S Analytical Ltd |
| 15.20 | The Role of Government<br>Lyndon Davies, LINK |
| 15.45 | Open Session Chaired by Ken Leiper, Glaxo Pharmaceuticals |
| 16.30 | End |

### Registration details

Please send your registration to:-

Dr E H Evans
University of Plymouth
Department of Environmental Sciences
Drake Circus
Plymouth PL4 8AA

Tel: 01752 233040
Fax: 01752 233035

| | | |
|---|---|---|
| Registration Fee (including lunch)<br>Members of Royal Society of Chemistry | £35.00 | ☐ |
| Registration Fee (including lunch)<br>Non-members | £45.00 | ☐ |
| Students/Pensioners, etc. | £25.00 | ☐ |

A small number of student bursaries are available from the Automatic Methods Group. Please contact Dr R. Narayanaswamy, c/o DIAS, UMIST, PO Box 88, Manchester M60 1QD.

